# Remarks on the Influence of Mental Cultivation and Mental Excitement upon Health

**Published:** 1845-04

**Authors:** 


*Remarks on the influence of Mental Cultivation and Mental Excitement upon Health.* By Amariah Brigham, M. D. Superintendant and Physician to the State Lunatic Asylum, Utica, New York. Third edition, 12mo. pp. 204. Lea & Blanchard, Philadelphia, 1845.

The first edition of this popular work was published some ten or a dozen years ago, and acquired for the author the reputation of a sound and original thinker. It is one of the few American works that have been deemed worthy of re-publication in Europe, where it has undergone several editions. We do not mean by this remark to be understood to admit that all the works of our countrymen that have not received such notice, are undeserving of the most extended circulation. We know the contrary—we could point out some in medicine, as well as in other sciences and general literature, which evince talents and acquirements on the part of their authors of the highest order; and of which Europeans have little, if any knowledge: but when a work does become thus distinguished, it is pretty conclusive evidence of its value.

We could wish that all intelligent parents and teachers would read this little volume. It would show them the folly and cruelty of the present preposterous system of education, in which the bodies of children, while in very infancy, are cramped and their health ruined by confinement, and their minds stultified by over taxed efforts to learn abstractions, before they have become acquainted with things, or acquired the simplest ideas.

